# The impact of higher education on youth employability in Kosovo: Key determinants affecting the interface between higher education provision and labor market needs

**DOI:** 10.12688/openreseurope.21616.2

**Published:** 2026-05-11

**Authors:** Xhavit Rexhaj, Arberesha Qerimi, Fidan Qerimi

**Affiliations:** 1 AAB College, Pristina, Pristina, 10000, Kosovo (Serbia and Montenegro)

**Keywords:** Curriculum flexibility, Graduate skills, Higher education, Kosovo, Labor market, University-industry collaboration, Youth employability.

## Abstract

**Objectives:**

This study aims to assess the impact of higher education on graduates’ perceived employability of young people through dimensions such as study programs in higher education (SPH), collaboration between the universities and businesses (CUB), teaching quality (TQ), flexibility of academic programs (FAP), and higher education infrastructure and resources (HEIR) by identifying factors that influence the interplay between the higher education provision and the demands of the labor market. In addition, differences in employability depending on the ownership of the higher education institution and the characteristics of the graduate profile and field of study are also analyzed.

**Methods:**

To achieve the research objectives, a quantitative method was used through a structured questionnaire, where 300 persons who have graduated in academic years 2019/20 – 2023/24 were surveyed.

**Findings:**

The results show a significant positive relationship between higher education and employability (R ^2^ = 0.367, p < 0.001), with labor market demands and program flexibility as the strongest predictors. Private institutions performed better in all dimensions, with the biggest difference displayed in infrastructure. The field of study also had a significant impact, with graduates in engineering and information technology fields showing the highest levels of employability.

**Novelty/improvement:**

This research aims to inform social partners about the ways to fill the gap between the supply of higher education and the needs of the labor market both at home and abroad through sustainable mechanisms, which would guide the provision of study programs.

## Plain Language Summary

This study explores how higher education in Kosovo affects young people’s ability to find suitable employment. It examines the connection between the skills acquired during university studies and the needs of the labor market. The research involved a survey of 300 graduates from both public and private institutions across Kosovo.

The results show that graduates from private colleges tend to report a stronger alignment between their studies and job requirements, while those from public universities face more challenges in translating academic knowledge into practical work skills. Key factors influencing employability include practical training, internships, and the relevance of curricula to market demands.

These findings highlight the need for universities to collaborate more closely with employers and to adapt study programs to better prepare students for real labor market conditions. The study contributes to understanding how education policy can reduce youth unemployment and enhance economic development in Kosovo.

## Introduction

The higher education system is considered to be one of the key factors for the competitive position of an economy, ^1^ where higher education is increasingly expected to engage with the challenges of the contemporary world. ^2^ ^,^ ^3^ To ensure the efficiency of the economy, quality education is one of the conditions that must be met because through improving tertiary education, it would be possible to move the economy to a higher level of development. ^4^ Therefore, higher education institutions should be considered as an element that not only stimulates and encourages the connection between learning, research and innovation, but also transfers knowledge to the economy. ^5^

Higher education is considered an important indicator that influences the improvement of youth employability, with many studies showing that young people who have a university degree tend to face lower unemployment rates compared to those without a tertiary level of education. ^6^ However, the increased participation in higher education in recent years has been accompanied by a challenging phenomenon, namely the mismatch between the skills of graduates and the needs of the labor market. ^6^ ^,^ ^7^ According to this, a university degree is not a sufficient criterion for employment, but what matters instead are the practical experiences of graduates, transferable skills and the compatibility of the graduate’s professional profile with market requirements.

In this situation, higher education institutions receive criticism for not preparing graduates for the real context involved in professional practices. ^8^ Therefore, employability turns into the main issues that drives the mission of higher education institutions. ^9^ The incorporation of employability as a key component in the mission of higher education prompts changes in teaching, learning and assessment methods, and in particular causing the adaptation of curriculum design and assessment methods in line with labor market demands. ^10^

When assessing quality of higher education, perspectives of graduates and other stakeholders should be taken into consideration, given the critical importance of the learning processes and competences of graduates. ^11^ The understanding and study of employability varies across disciplines, representing one of the missing links between the professional context and higher education institutions. ^12^ Higher education institutions should include employability competencies in the learning process to prepare students for the labor market and develop competencies that shape their readiness for work, ^13^ in particular since traditional theory-based teaching in higher education has led to a mismatch between the skills of graduates and the labor market needs.

Employability refers to the ability of graduates to obtain and maintain employment by possessing relevant knowledge, skills and competencies demanded by the labor market. However, employability should be distinguished from employment, which refers to the actual labor market status of an individual. In this study, employability is operationalized through self-reported employment outcomes, including the time required to find a job after graduation, the match between the job and the field of study, and other indicators related to graduates’ labor market integration.

The fact that a gap has emerged between competencies in higher education and labor market expectations has created a need for further research that studies this phenomenon. ^14^ According to studies, the skills gap is the lack of cognitive, technical, and interpersonal skills that are required by the labor market, but that are not sufficiently developed by higher education institutions. It is assessed that study programs are disconnected from labor market requirements, with universities promoting theoretical knowledge, at a time when the labor market values practical competencies and adaptation to a dynamic, constantly changing work environment. ^15^

Even though environmental factors cannot be controlled by higher education institutions, students’ competencies can be improved through teaching and learning experiences provided by universities. In relation to this, higher education institutions can take initiatives that would strengthen personal capital and career development to improve their employability. ^16^ This would reduce the gap that exists between the preparation of graduates and the unrealistic expectations of employers, who expect graduates to have applicable skills in different contexts immediately upon entering the labor market. ^17^ ^–^ ^19^ The implications of this gap in employability skills go beyond academic considerations with a direct impact on the employment opportunities offered to graduates, but also improving their professional longevity outlooks. The importance of addressing this phenomenon can reduce unemployment, increase productivity without stagnating the economy, and can contribute to the regional and national context. In this regard, understanding the complexity of the mismatch between the supply of higher education and the labor market demands can influence the proposal of viable solutions to reduce this gap. ^20^

The Kosovo context is a notable example of the challenges and incompatibilities between higher education and the labor market. At the outset of the third decade of this century, Kosovo saw a significant increase in participation in higher education, culminating at around 73% of young people aged 18-22 enrolled in higher education - one of the highest rates in Europe. ^21^ As a result, thousands of graduates tend to join the labor market annually. Another key tendency in Kosovo higher education in recent decades has been the rapid increase in the number of students enrolled in private higher education institutions (43 % of the total number of students in 2023 ^22^), which came as result of increased popular demand for higher education qualifications and a limited provision by public institutions. However, despite the growing percentages of persons with university qualifications, Kosovo continues to struggle with high unemployment rates (19.5% and low employment rates 38.6% ^23^) and a mismatch between higher education provision and employment.

As a result, this research aims to measure and analyze the impact of higher education on the employability of young people and to identify the factors that influence the mismatch between the educational provision and the demands of the labor market. A significant perspective into the impact of higher education on the labor market is obtained by measuring and analyzing the influence of the type of ownership of the HEIs as an independent variable. Subsequently, and based on the findings, the research aims to inform social partners (universities, industry, and authorities), about the ways to fill the gap between the supply of higher education and the needs of the labor market at home and in Europe as the main channels of employment of Kosovo graduates. The purpose is to come up with well-grounded recommendations and sustainable mechanisms, which would guide the relations between stakeholders and the design and provision of study programs.

## Literature review

Employability has become an important topic in higher education policy and research in recent years, with increasing emphasis on equipping graduates with the skills required to contribute effectively and productively to the economy. This would effectively reposition higher education as a means to an end, namely decent employment, rather than as an end in itself, namely the development of intellectually well-rounded individuals. ^24^ Higher education has recently been considered of little use in predicting the specific skills that are expected to be required for a job in the future, as a result, it is mandatory to stimulate the competencies of students in order to take advantage of opportunities for coping and creating self-employment opportunities after completing their studies. ^25^ This means that education cannot directly prepare graduates for the specific demands that the labor market may have, since labor market demands are mainly short-term and do not consider the general skills needed to cope with long-term developments and changes. It would be more opportune if the orientation of higher education institutions focused on increasing employability of graduates by preparing them to quickly learn specific workplace competencies. ^26^

The way in which the mismatch is interpreted depends on the understanding of theoretical knowledge and learning and the expectations for the function that higher education should have in relation to the labor market. The fact that higher education is heterogeneous and has different functions in relation to the labor market, where different jobs require different competencies according to sectors, the nature of the programs affects the applicability of competencies in the real labor market. ^27^

Many studies have addressed what students should learn and what they actually learn in relation to the demands of the job market. ^28^ ^,^ ^29^ Depending on the sectors and diversity of enterprises, workplace requirements also vary, as there are no uniform competencies required by all jobs, but general skills that are constantly discussed as required for the labor market. ^30^ The presence of technological innovation and organizational diversity creates a highly dynamic demand in the labor market, where the main challenge for graduates is to be up-to-date and ready to have a greater diversity of competencies than they had before. ^31^

A study dealing with graduate employability posits that labor market demands are related to observable and unobservable skills, defining these in an inclusive group as general skills that are transferable and useful for almost any job, with the exception of specific requirements that a job may have. ^32^ So, employability is considered to be related to the study programs, development of soft skills, and the necessary personal development in order to work within a team. ^25^

Despite the challenges that higher education institutions face, their goal is to prepare graduates with professional and general skills, however, various discrepancies and gaps have emerged between what is offered and what is required. For example, in a study by Nilsson, graduates in fields such as engineering and economics tended to have a wider range of positions in the labor market immediately after graduation, while graduates in professional programs such as law and medicine found the labor market to be more limited, with homogeneity of horizontal and vertical distribution for graduates in these fields identified. ^27^ As a result, higher education institutions can take various measures to improve employability despite the inconsistencies and problems of horizontal and vertical distribution, such as curriculum modifications, providing various trainings in terms of personal attributes, problem-solving skills, interpersonal skills and communication skills, job placement through internship opportunities, and providing learning from the experiences of successful former students, such as Alumni activities. ^33^ For this to be possible, teachers must be trained in these skills so that they are then competent in teaching students how to improve their entrepreneurial skills by introducing them more to the business environment.

Recent empirical research has further emphasized the growing importance of employability skills and the alignment between higher education and labor market demands. Studies conducted in different national contexts indicate that employability increasingly depends on the development of transferable skills, practical competencies and graduates’ ability to adapt to rapidly changing labor market conditions. ^20^ ^,^ ^34^ Contemporary research also highlights that the mismatch between higher education provision and labor market needs remains a persistent challenge, particularly when study programs fail to integrate practical experience, technological competencies and collaboration with industry partners. ^17^ ^,^ ^18^ Furthermore, recent evidence suggests that the relevance of university education for labor market integration is closely related to the extent to which curricula incorporate employability-oriented learning approaches and skill development strategies. ^35^ In this regard, recent studies emphasize that strengthening cooperation between universities and employers plays a crucial role in improving graduates’ employment outcomes and reducing the gap between acquired qualifications and labor market expectations. ^36^ ^,^ ^37^

Based on the theoretical arguments and empirical evidence discussed above, higher education institutions play a central role in shaping the competencies and skills needed for participation in the labor market. The literature suggests that the structure of study programs, the development of transferable skills, and the match between academic training and labor market needs significantly influence the employability outcomes of graduates. Studies show that employability increasingly depends on the ability of higher education systems to equip graduates with general and practical competencies that enable them to adapt to rapidly changing labor market conditions. ^20^ ^,^ ^34^ ^,^ ^35^ Based on these theoretical arguments, it can be expected that higher education plays a significant role in shaping graduates’ employability outcomes. Therefore, the following hypothesis is proposed:

**H**_**1**_: Higher education has a statistically significant impact on the perceived employability of graduates.

In addition to the general impact of higher education, institutional characteristics may also affect the employability outcomes of graduates. Differences in institutional governance, curriculum design, teaching approaches and collaboration with industry partners can influence how higher education institutions prepare students for labor market integration. ^17^ ^,^ ^18^ In this context, differences between public and private higher education institutions may lead to different perceptions of graduates regarding their employability. Therefore, the following hypothesis is formulated:

**H**_**2**_: There is a statistically significant difference in the level of perceived employability of graduates depending on the type of higher education institution (public or private).

The literature also highlights the role of individual characteristics of graduates in shaping employability outcomes. Factors such as field of study, academic background and other profile characteristics can influence how graduates assess their readiness for labor market participation and the extent to which their qualifications match employers’ expectations. ^27^ ^,^ ^32^ These differences may be reflected in variations in the level of perceived employability among graduates. In this context, the following hypothesis is proposed:

**H**_**3**_: There is a statistically significant difference in the level of perceived employability of graduates depending on the characteristics of the graduate profile.

Employability is also influenced by broader external factors related to labor market dynamics, technological changes, and employer expectations. Rapid technological developments and ongoing transformations in the structure of the labor market are increasingly influencing the competencies required by employers and the opportunities offered to graduates for professional integration. ^31^ ^,^ ^36^ ^,^ ^37^ These external factors may also influence how graduates perceive their employability. Based on these theoretical arguments, the following hypothesis is proposed:

**H**_**4**_: External employability factors outside higher education have a statistically significant impact on the perceived employability of graduates.

**Figure 1.**
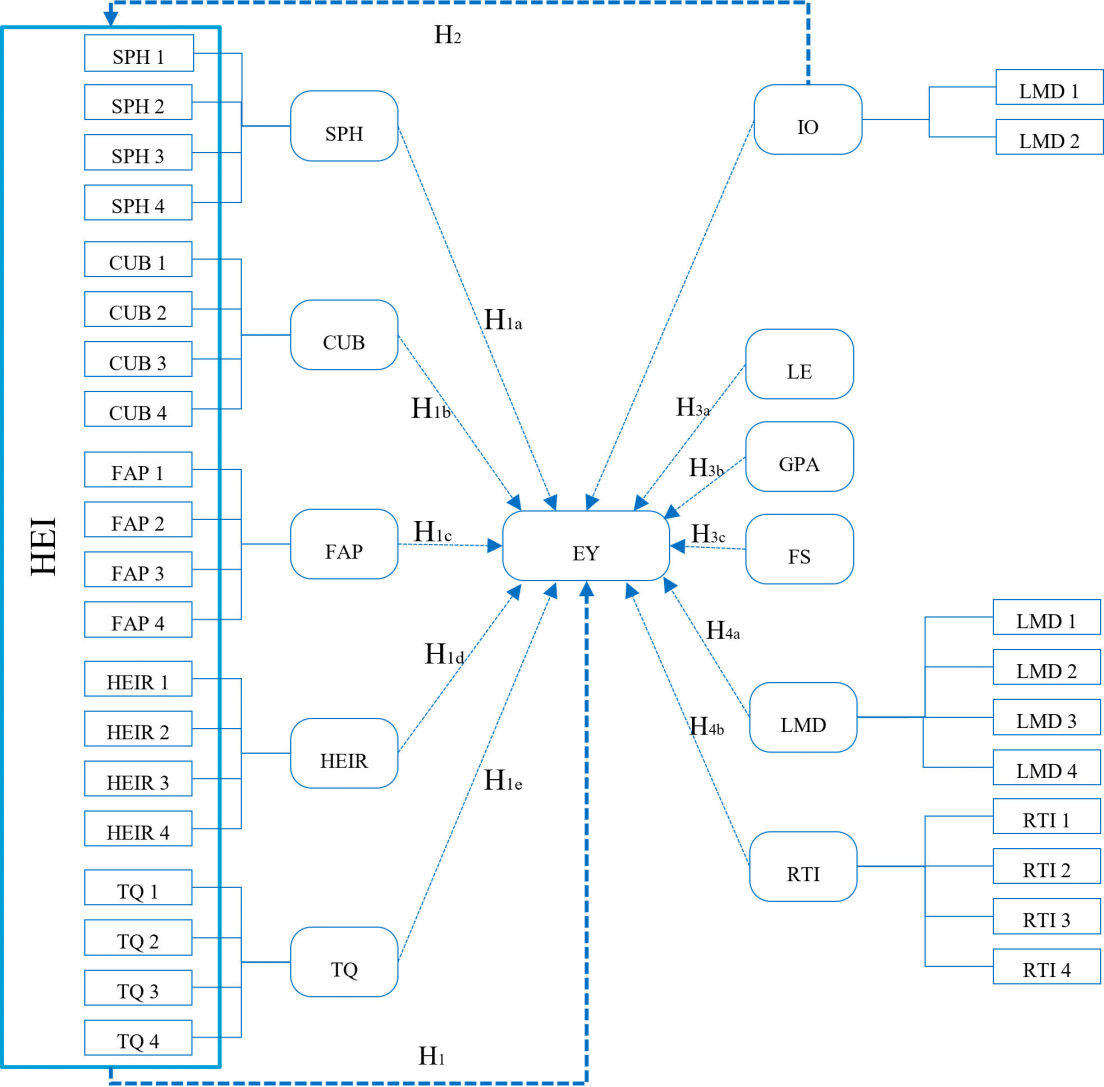
Framework of research.

To see the relationship between the variables, refer to the framework below Figure 1:

### Kosovo context

In Kosovo, there is a vivid gap between the labor market demands and higher education provision, a fact that negatively affected the employability of young people. This discrepancy is assessed as a failure of educational structures to function in accordance with the socio-economic needs of the country, where higher education institutions continue to focus mainly on theoretical content without including practical components to provide students with skills to face challenges of the labor market. Curricula in the large part of the programs offered by higher education do not reflect a technological, economic and social perspective. In addition, this gap is further deepened, due to the lack of institutionalized mechanisms of cooperation between higher education and the labor market, such as advisory/industrial boards, and as a result, graduates do not possess applicable and transferable skills in real work contexts. ^38^ This mismatch between labor market demand and higher education is a proven phenomenon where despite the growth in the number of higher education institutions and the increase in the number of graduates, the labor market is failing to absorb them, with a fairly high proportion of graduates remaining unemployed or even employed in fields unrelated to their qualifications, proving a functional gap between the needs of the labor market and the education system. ^39^

**Figure 2.**
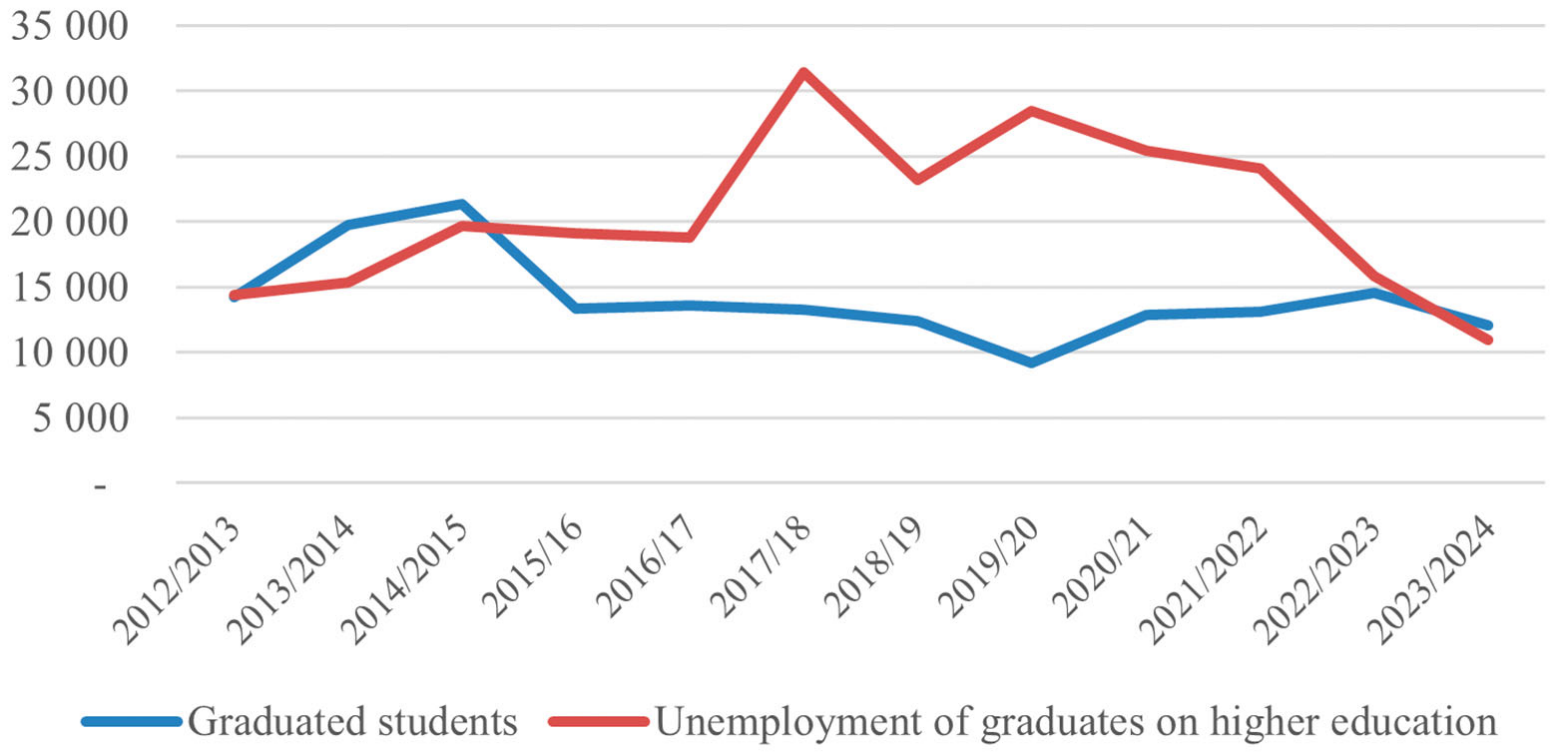
Number of graduated students and unemployed graduates from HE. Source: Kosovo Agency of Statistics, 2025.

The analysis of the trend of the number of graduates and their employability presented in Figure 2 shows that in the period 2012/2013 to 2023/2024, the number of active students increased continuously until 2016/2017, reaching 123 243 students, ^40^ after which a sharp decline began, dropping to 71 117 ^41^ in the 2023/2024 academic year. This change indicates a shift from massive expansion to a narrowing of the student base, which may be related to demographic factors, emigration, saturation of the demand for higher education, or a lack of motivation for higher education due to high levels of graduate unemployment. The number of graduates reached an early peak in 2014/2015 with 21 364 graduates but then fell significantly and remained stable at a lower average. At the same time, unemployment among higher education graduates increased sharply, peaking in 2017/2018 with 31 436 unemployed, more than the number of graduates that year, which signals a build-up of unemployment from previous years and an increased mismatch with the labor market. After 2021/2022, unemployment gradually decreased, which could be related to the reduction in educational supply, improved market adaptation, or even graduate emigration. The latter could be linked to the EU visa liberalization for Kosovo in 2023 and introduction of working visas for Kosovo labor force to Western Countries, as a result of which Kosovo has been losing between 25 000 and 40 000 inhabitants annually for the period 2019-2024. ^42^ Overall, the data reflects a cycle of transition from quantitative expansion to an effort for structural adjustment and higher effectiveness of higher education in relation to market demands.

The World Bank and UNDP reports emphasize that higher education in Kosovo is not making a sufficient contribution to improving employability. This is related to the lack of cooperation and student internships with industry to develop transversal competencies, as a result of which graduates often do informal work, or are looking for opportunities to migrate for better opportunities. ^43^ ^,^ ^44^

In the same context, the European Training Foundation (ETF) emphasizes that policy and operational measures should be taken to create a more integrated system of education and employment to enable the harmonization of academic content with labor market requirements, defining the function of higher education as a catalyst for economic development. ^45^

## Methods

To achieve the research objectives, a quantitative method was used, where the population was students and graduates in five years of the period 2019/20-2023/24 which are shown on Table 1, data obtained from the Kosovo Agency of Statistics. The sample for this study consists of 300 graduates, selected through simple random selection, in accordance with the real distribution of graduates in Kosovo during the years 2019/20-2023/24. The questionnaire was distributed electronically through alumni networks, university communication channels and professional social media groups targeting graduates who completed their studies during the period 2019/20–2023/24. In total, 360 graduates were contacted, of which 300 completed the questionnaire, resulting in a response rate of 83.3%. To reduce potential non-response bias, the distribution of respondents was monitored to ensure proportional representation of graduates from both public and private higher education institutions. Out of a total of 64,784 graduates, 42% belonged to private institutions and 58% to public ones. Therefore, the sample was also divided proportionally with 127 from the private sector and 173 from the public sector, ensuring fair representation for statistical analysis.

**Table 1.** Graduate students from Kosovo higher education private and public institutions between 2019-2024 (KAS).

	2019/20	2020/21	2021/22	2022/23	2023/24	Total	Percentage	Sample
Private	2 270	6 094	5 257	7 665	6 070	27 356	42%	127
Public	6 216	7 205	7 892	7 272	8 843	37 428	58%	173
				Total		64 784	Total	300

*Source: Kosovo Agency of Statistics, 2025.*

### Informed consent procedures

**Figure 3.**
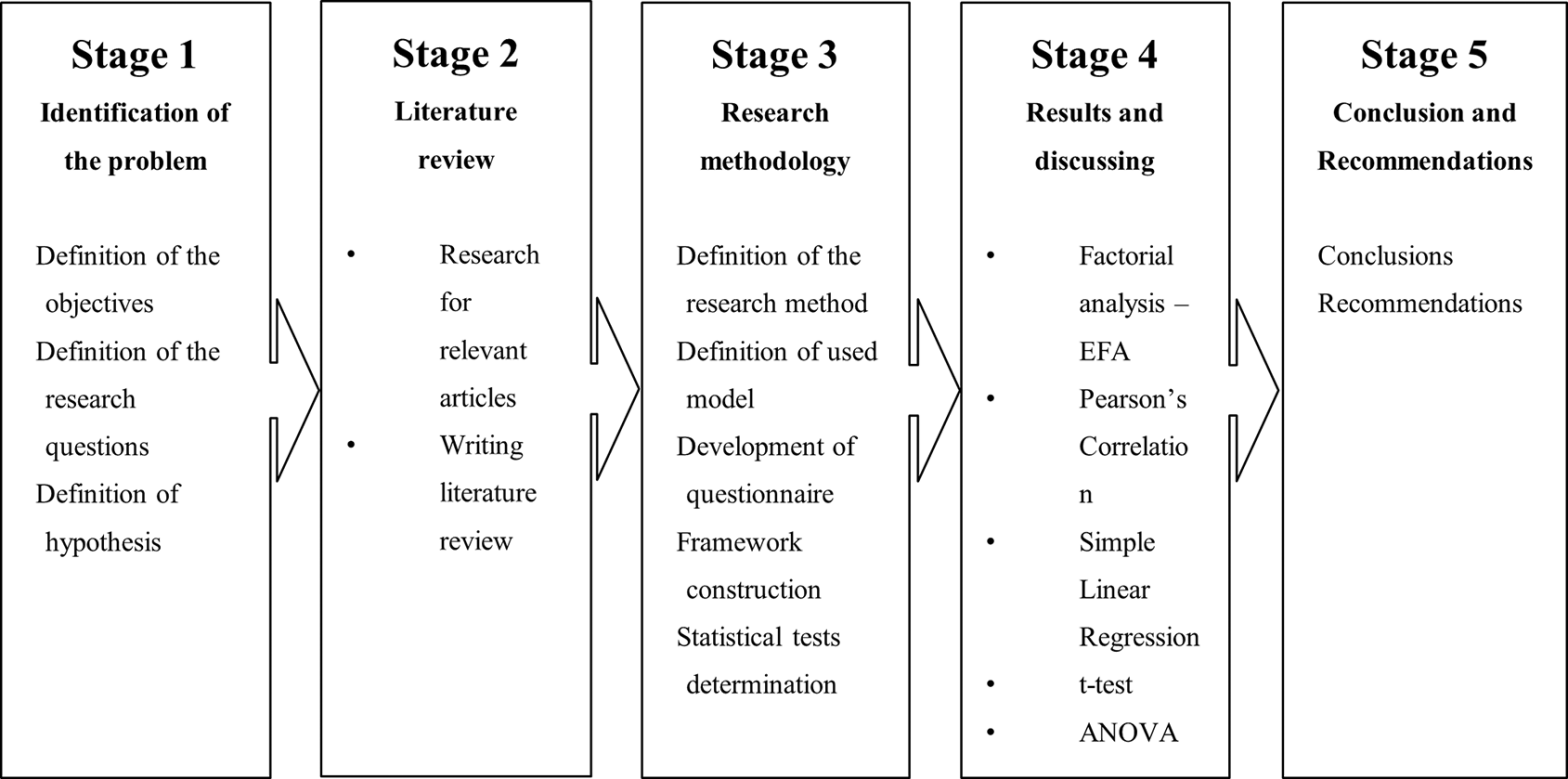
Flowchart of research methodology.

The survey instrument was structured and divided into three sessions. The measurement dimensions and items included in the questionnaire were developed by the authors based on the existing literature on graduate employability and the alignment between higher education and labor market demands. The items were designed to capture latent constructs related to study programs, teaching quality, university–industry collaboration, flexibility of academic programs, infrastructure and resources, and graduates’ perceived employability. Before the main data collection, the questionnaire was pretested with a small group of graduates (n = 20) to ensure clarity of wording and relevance of the items. Minor adjustments were made based on the feedback obtained during this pilot phase. The first session contained demographic questions and Graduate Profile Characteristics, the second session included five measurement dimensions for higher education (study programs (SPH), teaching quality (TQ), university-industry collaboration (CUB), flexibility of academic programs (FAP), infrastructure and resources (HEIR) constructed according to the Likert scale and part of the third session was the employability of youth (EY) is measured through graduates’ perceptions regarding their readiness for the labor market and the alignment of their skills with employer requirements. Since the data were collected through Likert-scale responses in the questionnaire, this variable reflects perceived employability rather than the actual employment status of respondents. The questionnaire items were measured using a five-point Likert scale ranging from 1 (strongly disagree) to 5 (strongly agree). Each dimension was measured through multiple items designed to capture the respective latent construct. The coding scheme used in the dataset follows the variable abbreviations applied in the study (SPH, CUB, TQ, FAP, HEIR, LMD, RTI, EY). Missing responses were minimal and were handled through listwise deletion during the statistical analysis to ensure consistency of the regression models. For transparency and replicability, the full questionnaire including the exact wording of all items is provided in Ref. 46. Figure 3 presents the research methodology.

To determine the distribution of data, the Kolmogorov Smirnov test and the Shapiro Wilk test were used, an important condition for determining the tests. According to the test results, the significance value is p>0.05, which means that we have a normal distribution of data and the condition for using the Pearson correlation and parametric tests for comparing means is met. Also, the internal consistency of the measuring instrument was measured through Cronbach’s alpha coefficient, where the value α=.875 argues that the questionnaire has acceptable reliability. So, this test is done to assess whether the questions of a measurement scale function as a connected group and accurately measure a single construct. The calculation of this coefficient was done through the following equation:

$$\alpha =\frac{k}{k-1}(1-\frac{\sum_{i=1}^{k}\sigma_{i}^{2}}{\sigma_{total}^{2}})$$ (1)

Also, exploratory factor analysis (EFA), a statistical technique of multiple variables, is used to reduce the number of variables that are found in relation to each other to a smaller number of significant variables and independent of each other. Factor analysis includes different techniques, but at the same time they are related to each other. The first test applied is Kaiser Meyer Olkin – KMO and Bartlett’s Test of Sphericity. KMO argues the suitability of the selected sample for factor analysis, where the KMO level should be above 0.50, the higher the coefficient, the better the data set is to do factor analysis. While through and Bartlett’s Test of Sphericity shows whether the data set is suitable and that there is a proper theoretical basis for factor analysis. Such measurements are made through the following equations:

$$KMO=\frac{\sum_{i\neq j}r_{ij}^{2}}{\sum_{i\neq j}r_{ij}^{2}+\sum_{i\neq j}p_{ij}^{2}}\;x^{2}=-\left(n-1-\frac{2p+5}{6}\right)*ln\left|R\right|$$ (2)

Through communality, the contributing variables in the construction of the latent structure are assessed and to identify which ones represent weak loadings for interpretation. Thus, it measures the degree to which a variable is represented by the common factors of the factorial model. The following equation represents the mathematical calculation of communality.

$$h_{i}^{2}=\sum_{j=1}^{m}\lambda_{ij}^{2}$$ (3)

Through correlation analysis, the relationship between the dimensions that measured variables of the higher education dimensions (SPH, CUB, TQ, FAP, HEIR, LMD, RTI) and youth employability (EY) were tested. The Pearson correlation coefficient measures the direction and strength of the linear relationship between the variables treated in the paper, where depending on the value of r, we determine the level of the relationship. The Pearson correlation was calculated through the following equation:

$$r=\frac{\sum \left(x_{i}-\bar{x})(y_{i}-\bar{y}\right)}{\sqrt{\sum {\left(x_{i}-\bar{x}\right)}^{2}}*\sqrt{\sum {\left(y_{i}-\bar{y}\right)}^{2}}}$$ (4)

The *F* statistics are used in the regression model to measure whether the variation between groups is statistically greater than within them. So, through the equation below, it is tested whether the regression model is valid as well as to measure whether the groups in the ANOVA differ for the dependent variable:

$$F=\frac{MSR}{MSE}=\frac{\frac{SSR}{k}}{\frac{SSE}{\left(n-k-1\right)}}$$ (5)

To measure what percentage of the variance of the dependent variable is explained by the independent variable, R Square was used, while avoiding overfitting the model when many variables are added, Adjusted R Square was used. Calculations were made based on the following equations:

$$R^{2}=\frac{SSR}{SST}\;;\;Adjusted\;{\bar{R}}^{2}=1-\left(\frac{\left(1-R^{2})(n-1\right)}{n-k-1}\right)$$ (6)

The important test is the Durbin Watson test, through which we tested whether autocorrelation exists in our model. The test values should be between 1.5 and 2.5 to assess that the model does not have autocorrelation problems. This measurement is made through the following equation:

$$DW=\frac{\sum_{i=2}^{n}{\left(e_{i}-e_{i-1}\right)}^{2}}{\sum_{i=1}^{n}e_{i}^{2}}$$ (7)

The equation below represents the linear regression, which was used to measure the impact of higher education on employability. Beta, as a standardized coefficient, was used to measure the relative impact of each independent variable. So, through B, we measured which factors of higher education have the greatest impact on the employability of young people:

$$Y=B_{0}+B_{1}X_{1}+B_{2}X_{2}+\cdots ++B_{n}X_{n}\;;\;\beta =B*\frac{\sigma_{x}}{\sigma_{y}}$$ (8)

The t-test is used to investigate the difference between two sample groups in terms of means. The test determines whether there is a significant difference between the mean of one group and the mean of the other group. In this case, through the equation below, we tested whether students’ perceptions regarding employability and the measurement dimensions of higher education differ between private and public higher education institutions in Kosovo:

$$t=\frac{{\bar{x}}_{1}-{\bar{x}}_{2}}{\sqrt{\frac{s_{1}^{2}}{n_{1}}+\frac{s_{2}^{2}}{n_{2}}}}$$ (9)

Analysis of variance is used to test the hypothesis about whether there is a significant difference between two or more means. One Way ANOVA is a simple analysis of variance and consists of two variables. One of these variables has categorical characteristics (independent variable) and the other variable has metric characteristics (dependent variable). In analysis of variance, the F value is used to test the hypothesis through the following equation:

$$F=\frac{\frac{SS_{betwwen}}{df_{between}}}{\frac{SS_{within}}{df_{within}}}$$ (10)

When a significant difference is found between groups in the analysis of variance, the Post Hoc test is important to see which group the difference originates from. The test used to identify where the difference originates within the Post Hoc tests is the Tukey HSD test. Through this test, it was identified which areas have higher employability, by grouping them into 3 subsets. This measurement was made through the following equation:

$$HSD=q*\sqrt{\frac{MS_{within}}{n}}$$ (11)

## Results and discussion

The demographic data in Table 2 represents a clear distribution of educational attainment by age group, reflecting the actual profile of graduates in Kosovo over the past five years. The sample is dominated by individuals aged 24–32 (over 70%), where a shift from Bachelor to master’s degrees is evident, aligned with increasing labor-market demands for advanced qualifications. The 21–23 age group consists mainly of Bachelor graduates, while those aged 27–32 are almost entirely Master’s graduates, indicating professional consolidation and academic maturity. Doctoral degrees appear only after age 33, with the highest concentration in the “36 and above” group, consistent with the timeline required to complete third-cycle studies.

**Table 2.** Demographic data.

Age Group (Years)	Bachelor’s Degree	Master’s Degree	Doctorate (PhD)	Total	%
21–23	66	0	0	66	22.0
24–26	36	67	0	103	34.3
27–29	6	45	0	51	17.0
30–32	0	56	0	56	18.7
33–35	0	6	3	9	3.0
36 and above	0	6	9	15	5.0
Total	108	180	12	300	100.0

*Source: Authors’ calculations based on survey data.* ^39^

**Figure 4.**
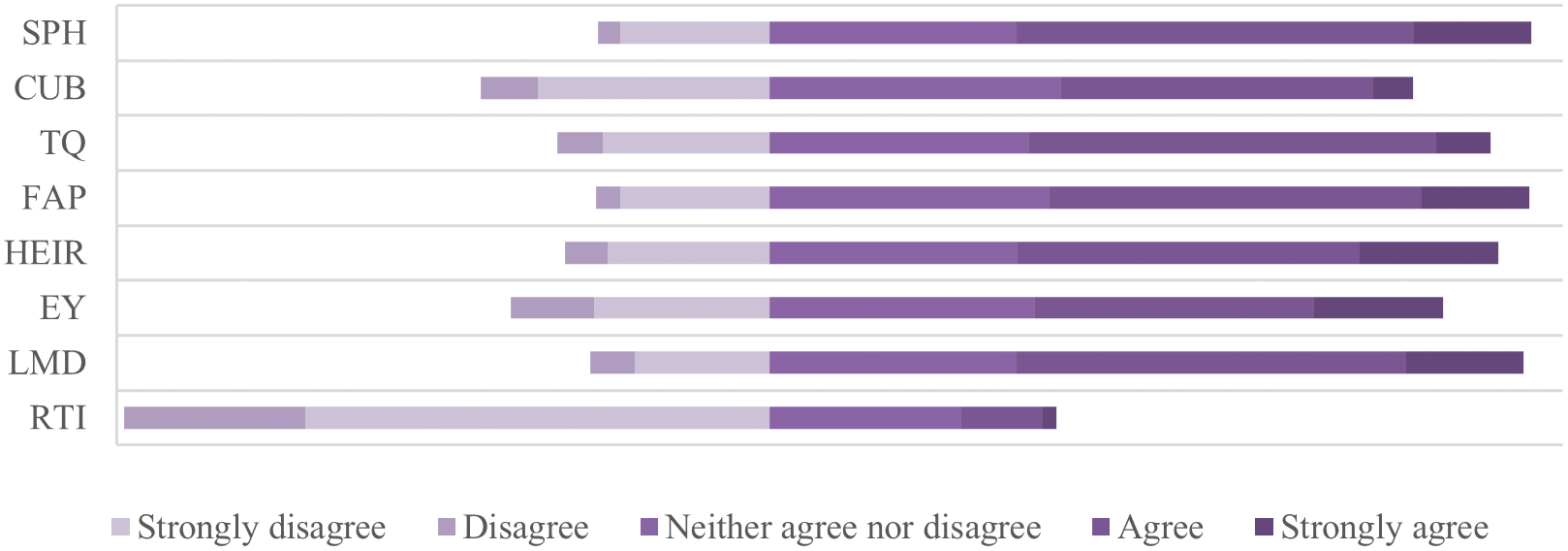
Degree of compliance for variables. Source: Authors’ calculations based on survey data. ^46^

Figure 4 shows perceptions of graduates from HEIs in Kosovo regarding the dimensions influencing the relevance of higher education to the needs of the labor market. Based on the obtained data, show fragmented empirical analyses, where some of the dimensions show positive results from graduates, and some others reflect to the gaps and systematic challenges, which reflect gaps and systematic changes in the functioning of higher education in Kosovo and are associated with differences in graduates’ perceived employability. Even though study programs are generally assessed as partially compatible with labor market requirements, the results indicate uneven alignment across different dimensions of higher education. In particular, the relatively lower evaluations of university and business cooperation and the role of technology and innovation suggest that important elements of higher education provision remain insufficiently connected to labor market expectations. These findings indicate that the alignment between higher education objectives and labor market needs remains limited in several key areas. Cooperation between universities and businesses highlights fundamental problems, which report systemic weaknesses of higher education. Cooperation between universities and businesses turns out to be one of the weakest components, reflecting a functional lack of connection with industry and the institution, limiting students to have the opportunity to apply theoretical knowledge in practice, in this case creating a gap between theoretical knowledge and applicable skills in the reality of the labor market.

The most worrying result concerns the role of technology and innovation as the most critical indicator in terms of overall compatibility, data that show a lack of integration of technology and innovation in the content of study programs, presenting a large gap between the content of study programs and the characteristics of the labor market, which are changing exponentially. Regarding employability, the findings also reveal a fragmented pattern. Graduates’ responses indicate that higher education is perceived as facilitating labor market entry for some respondents, whereas others report the need for additional training after graduation, employment outside their field of study, or employment in positions below their qualification level. These patterns reflect variation in graduates’ self-reported employment outcomes and suggest that the alignment between higher education provision and labor market expectations remains uneven. The findings further indicate that 46% of respondents reported longer periods before securing employment after graduation, while 58% reported need for additional training as a condition for obtaining suitable employment. These self-reported outcomes support the argument that a mismatch exists between higher education provision and labor market needs. The conservative nature (in particular among the public) higher education institutions and the resulting slow response to rapidly changing trends in the new technologies and the labor market, is a call for alarm for education authorities to review the higher education responsiveness and provision in Kosovo. It should be added that most of the graduates were first engaged in a job other than their field of study (49%), requiring lower qualifications than their degree (41%) and therefore being underpaid when compared to their university qualification (38%). Moreso, despite their formal ‘overqualification’ they had to undergo additional training to enjoy more adequate earnings in their job place.

These indications speak in favor of universities paying more attention to the needs and trends in the labor market and engaging in more flexible and responsive study programs for their students and future graduates. This is only possible by engaging in closer dialogue and partnerships with the industry and providing shorter, more flexible, and most notably tailor-made training and study programs/qualifications for their students and industry partners In general, the results show that some dimensions tend to align with labor market demands, while *university cooperation with industry* and *the inclusion of technology and innovation* present critical results, a duality makes it necessary to reform higher education policies by orienting towards an integrated, innovative and practical model, which may be relevant for improving graduates’ perceived employability and their self-reported labor market integration outcomes.

**Figure 5.**
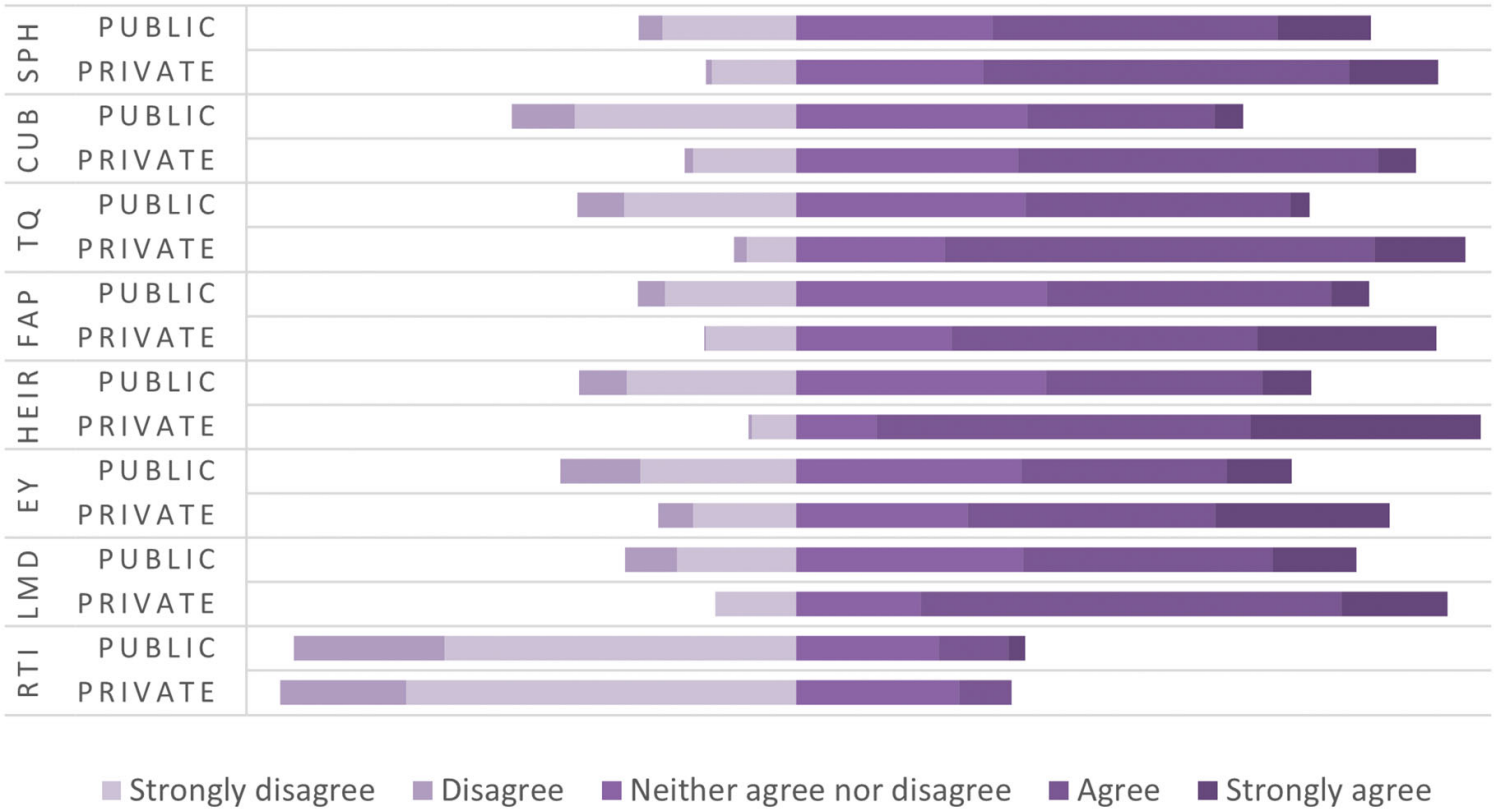
Degree of compliance for variables - comparison between private and public institutions. Source: Authors’ calculations based on survey data. ^46^

Figure 5 presents the comparison between private and public higher education institutions regarding the perceptions of graduates regarding the dimensions influencing the relevance of higher education related to the needs of the labor market. In the first dimension, that of higher education programs (HE), private institutions perform better than public ones. Graduates from private institutions had a higher tendency of compliance with the study programs, assessing them as more up to date, oriented towards labor market requirements, and supporting the development of practical skills. On the other hand, graduates from public institutions had neutral or negative attitudes, emphasizing the mismatch of the curriculum with labor market requirements and the lack of inclusion of the practical work component in academic curricula. The second dimension represents the cooperation of higher education institutions with industry (CUB), where the difference between private and public institutions is even more evident than in programs dimension (HE). Private institutions were assessed to be more involved in creating and linking collaborations with industry by transferring theoretical skills into practice. In public institutions, a significant lack of cooperation with industry and a low level of international cooperation for training opportunities and capacity development were identified, a fact that has influenced the mismatch of higher education with market demands. Teaching quality (TQ) is another dimension addressed within the framework of higher education, a dimension in which private education institutions yet again stood in a more favorable position than the public ones. Graduates of public institutions stated that teaching methods are oriented more in the theoretical aspect and less in solving concrete problems and lacked the professional orientation of students. In this context, students of private institutions stated that teaching methods are contemporary, with teachers involved in research regarding labor market demands and bringing the current labor market into the teaching process. The flexibility of academic programs (FAP) is another dimension perceived more positively by graduates of private institutions through providing the opportunity to choose appropriate modules by adapting their skills to market demands, recognizing practical experience as part of academic credits and including courses on new technologies. Meanwhile, public institutions, according to graduates’ perceptions, had a more negative approach, assessing that public institutions have a bureaucratic structure, are not aligned with the demands of the labor market, and showing significant lack of flexibility to adapt to individual student needs. If we refer to the infrastructure and resources of higher education (HEIR), we find that public institutions have a serious problem in this context, as a result of the lack of investment in laboratories and a modern environment for practical learning, lack of access to advanced resources such as electronic libraries, scientific databases, professional software and lack of orientation to offer courses or certifications to help students be more competitive in the market. In this line, private institutions resulted in infrastructure investments oriented towards quality culture.

These findings posit that private institutions are more oriented towards preparing students in line with the demands of the labor market than public ones. This difference reveals the need for substantial reform of public institutions in terms of updating curricula, infrastructure investments, increasing the flexibility of academic programs and the quality of teaching, to eliminate the gap between higher education and labor market demands, and avoid consequences on employability.

Figure 5 shows also the comparability between private and public institutions in terms of employability (EY), matching labor market requirements (LMD) and the role of technology and innovation (RTI). The results indicate that graduates from private institutions more positively evaluate the usefulness of their education for labor market integration, whereas graduates from public institutions report a more fragmented transition from higher education to employment. Even in labor market requirements, the perception is more positive among graduates of private institutions than public ones, where public institutions show a lack of alignment of academic curricula with the dynamic nature of the labor market, mainly in the practical skills required by employers. Interesting results for both types of institutions were obtained regarding the role of technology and innovation in higher education, viewing them as ignored or marginalized factors in academic curricula, further proving that preparing students for market needs in terms of technology is a challenge throughout the higher education system.

Factor analysis was used as a statistical technique of multiple variables to reduce the number of variables that are found in relation to each other to a smaller number of significant variables and independent of each other. For the assessment of the adequacy of the data, the statistical indicators Kaiser-Meyer-Olkin (KMO) and Bartlett’s Test of Sphericity come into consideration. Bartlett’s Test of Sphericity tests the possibility of testing high correlations between at least a part of the variables in the correlation matrix, while KMO is an index that compares the size of the observed correlation coefficient with the size of the partial coefficient. The statistics obtained in Table 3, with the KMO index of 0.889, indicate sample adequacy, which means that the relationships between the variables are sufficient to obtain reliable factors in the analysis. Also, based on the values (Chi-Square = 1308.312; df = 28; p < .001) of Bartlett’s Test, we confirm that there is a significant correlation between the variables and as a result the data is suitable for exploratory factor analysis (EFA).

**Table 3.** KMO and Bartlett’s Test.

KMO and Bartlett’s Test		
Kaiser-Meyer-Olkin Measure of Sampling Adequacy.		0.889
Bartlett’s Test of Sphericity	Approx. Chi-Square	1308.312
	Df	28
	Sig.	0.000

*Source: Authors’ calculations based on survey data*. ^46^

Communalities statistics are an indicator of the percentage of variance of each variable that is explained by the factorial structure. Since all values are greater than 0.5, we say that the suitability of the variables in this factorial model is evident and one of the key criteria for model acceptance is met. Referring to the Rotated Component Matrix, the factorial structure is statistically acceptable and provides support from high Communalities and clarity of factor loadings. The variables SPH, CUB, TQ, FAP, HEIR, EY, LMD, have strong loadings and are representatives of a latent factor that argues that the model is adequate. Also, according to the Varimax rotation, no correlation has been identified between the factors, which means that they are orthogonal, which means that there is no overlap of information between the factors.

**Table 4.** Communalities and Rotated Component Matrix ^a^.

			Rotated Component Matrix ^a^		
**Communalities**			**Component**		
	**Initial**	**Extraction**		**1**	**2**
SPH	1.000	0.583	SPH	0.712	0.277
CUB	1.000	0.724	CUB	0.811	0.258
TQ	1.000	0.718	TQ	0.829	0.174
FAP	1.000	0.772	FAP	0.874	0.089
HEIR	1.000	0.744	HEIR	0.861	0.046
EY	1.000	0.713	EY	0.758	-0.372
LMD	1.000	0.677	LMD	0.714	-0.408
RTI	1.000	0.793	RTI	0.161	0.876
Extraction Method: Principal Component Analysis.			Extraction Method: Principal Component Analysis. Rotation Method: Varimax with Kaiser Normalization.		

*Source: Authors’ calculations based on survey data.* ^46^

^a^ Rotation converged in 3 iterations.

To analyze the relationship between the independent and dependent variables, Pearson Correlation was used, where the correlation of SPH with EY (r = .425; p < 0.01) is a moderate positive linear relationship, which means that the structure of study programs is positively associated with graduates’ perceived preparation for work. According to the values (r = .468; p < 0.01), the correlation between CUB and EY is a moderate positive linear relationship, where cooperation between universities and businesses improves the cooperation between universities and businesses is positively associated with graduates’ perceived employability and more favorable self-reported employment outcomes. The correlation between TQ and EY (r = .513; p < 0.01) is estimated to be a moderate positive relationship, which means that the quality of teaching is positively associated with graduates’ perceptions of their preparation labor market integration. The correlation (r = .606; p < 0.01) between FAP and EY and (r = .625, p < 0.01) between LMD and EY is indicative of a strong positive linear relationship between the variables. So, the more flexible academic programs are perceived to be, the higher graduates’ perceived employability tends to be. According to the values (r = .546; p < 0.01) between HEIR and EY there is a moderately strong relationship, indicating that more positive professional preparation. The RTI variable did not show a statistically significant association with EY in the regression model (p = 0.18) and was therefore not retained as a significant predictor in the interpretation of the regression results.

**Table 5.** Correlation matrix.

	SPH	CUB	TQ	FAP	HEIR	LMD	RTI	EY
SPH	1							
CUB	.576**	1						
TQ	.548**	.688**	1					
FAP	.589**	.679**	.711**	1				
HEIR	.514**	.727**	.705**	.715**	1			
LMD	.403**	.390**	.443**	.539**	.556**	1		
RTI	.276**	.248**	.213**	.204**	.147*	-0.075	1	
EY	.425**	.468**	.513**	.606**	.546**	.625**	-0.078	1

**Note:** SPH = Study programs in higher education, CUB = Collaboration between the university and businesses, TQ = Teaching quality, FAP = Flexibility of Academic Programs, HEIR = Higher Education Infrastructure and Resources, LMD = Labor market demands, RTI = Role of technology and innovation, EY = Employability of youth.

*Source: Authors’ calculations based on survey data.* ^46^

According to the results of the simple linear regression, R = .606 shows that there is a strong relationship between HE and employability, while R ^2^ = .367 means that 36.7% of the variance in youth employability is described by the HE measurement dimensions. Adjusted R ^2^ = .365 proves that the model is stable. Another important indicator is the Std. Error of the Estimate (Std. Err = 0.51898) which proves that the predictions are accurate in relation to the degree of data variation. The F value (1, 298) = 172.967, and sigF < 0.05 confirms that the model is statistically significant and that the association between higher education and graduates’ perceived employability is significant. The Durbin-Watson test (2.073) shows that the model does not have an autocorrelation problem and that the assumption of independence of errors is met.

**Table 6.** Model summary ^b^.

Model	R	R Square	Adjusted R Square	Std. Error of the Estimate	Change Statistics					Durbin- Watson
					R Square Change	F Change	df1	df2	Sig. F Change	
1	.606 ^a^	0.367	0.365	0.51898	0.367	172.967	1	298	0.000	2.073

^a^ Predictors: (Constant), Higher education.

^b^ Dependent Variable: Employability of youth.

*Source: Authors’ calculations based on survey data*. ^46^

Based on the model below, the value β _0_ = 1.217, describes the expected value of youth employability when the value of HE components is zero, a one-unit increase in HE is associated with a 0.600-unit increase in EY within the estimated model. Std. Error = 0.046, t = 13.152 and p < 0.01 emphasize that the model is statistically highly. The regression results show that higher education is a significant predictor of graduates’ perceived employability. More specifically, more positive evaluations of the quality and relevance of higher education are associated with higher levels of perceived readiness for labor market integration and more favorable self-reported employment outcomes.

Model:

$$y_{\left(EY\right)}=\;y=\beta_{0}+\beta_{1}*x_{1}+\varepsilon \;1.217+0.600*x_{\left(HE\right)}$$ (12)

**Table 7.** Coefficients. ^a^

Model		Unstandardized Coefficients		Standardized Coefficients	t	Sig.
		B	Std. Error	Beta		
1	(Constant)	1.217	0.155		7.876	0.000
	Higher education	0.600	0.046	0.606	13.152	0.000

*Source: Authors’ calculations based on survey data.* ^46^

^a^ Dependent Variable: Employability of youth.

To measure the individual impact of each HE descriptive factor on employability, seven separate simple regression models were conducted with employability as the dependent variable and independent variables (SPH, CUB, TQ, FAP, HEIR, LMD, RTI) tested separately in regression analysis.

According to the simple linear regression results, SPH explains 18.0% of the variance in graduates’ perceived employability (R ^2^ = 0.180, β = 0.422, p < 0.05). Similarly, CUB explains 21.9% of the variance (R ^2^ = 0.219, β = 0.372, p < 0.05), TQ explains 26.3% (R ^2^ = 0.263, β = 0.466, p < 0.05), FAP explains 36.7% (R ^2^ = 0.367, β = 0.493, p < 0.05), HEIR explains 29.8% (R ^2^ = 0.298, β = 0.404, p < 0.05), and LMD explains 39.0% (R ^2^ = 0.390, β = 0.527, p < 0.05). In contrast, RTI did not show a statistically significant association with EY in the regression model (p = 0.18). The regression results indicate that LMD, FAP, HEIR, and TQ show the strongest statistical associations with graduates’ perceived employability in the estimated model. RTI did not show a statistically significant relationship with EY in the regression model, despite its theoretical importance in literature. This suggests that, within the current sample. Technology and innovation were perceived as important in the broader context of higher education, but they did not emerge as a significant predictor of perceived employability when empirically examined as an individual dimension. This discrepancy between the theoretical importance of technology and innovation and their empirical performance in the model may indicate that, although digital and technological competences are widely highlighted in the literature as key drivers of employability, their integration into higher education curricula and teaching practices in the Kosovo context may still be limited. In this sense, the non-significant empirical result may reflect a gap between the expected role of technological competences in theory and their practical inclusion in study programs.

**Table 8.** Simple linear regression for dimensions. ^a^

Variables	β	R	R Square	Adjusted R Square	t	Sig.	Std. error	Durbin Watson
SPH	0.422	0.425	0.180	0.178	8.099	0.00	0.053	1.679
CUB	0.372	0.468	0.219	0.216	9.130	0.00	0.041	1.888
TQ	0.466	0.513	0.263	0.26	10.304	0.00	0.045	2.013
FAP	0.493	0.606	0.367	0.365	13.150	0.00	0.037	1.99
HEIR	0.404	0.546	0.298	0.296	11.259	0.00	0.036	2.159
LMD	0.527	0.625	0.390	0.388	13.812	0.00	0.038	2.004
RTI	-0.072	0.078	0.006	0.003	-1.3430	0.18	0.054	1.577

^a^ Dependent Variable: EY.

**Note:** SPH = Study programs in higher education, CUB = Collaboration between the university and businesses, TQ = Teaching quality, FAP = Flexibility of Academic Programs, HEIR = Higher Education Infrastructure and Resources, LMD = Labor market demands, RTI = Role of technology and innovation, EY = Employability of youth.

*Source: Authors’ calculations based on survey data*. ^46^

Table 9 presents the averages of the variables treated in the paper comparing institutions by ownership. The average SPH for private is x=3.62 and SD=0.55 and public x=3.40 and SD=0.69, showing an average difference of 0.22 in favor of the private sector, a not very large difference but the high consistency in the assessments in the private sector proves that the study programs are evaluated more positively, are more standardized for students. The private sector has problems with fragmentation of study programs, lack of updating and lack of practical integration. The average CUB for privates is x=3.43 and SD=0.55 and public x=3.39 and SD=0.69, showing an average difference of 0.57 in favor of private institutions where students graduating from private institutions report that the university’s cooperation with industry is stronger, suggesting a more practical orientation. According to statistics, public universities were found to be more bureaucratic and without flexible mechanisms for cooperation with industry. The average TQ for privates is x=3.74 and SD=0.47 and public x=3.05 and SD=0.71, with an average difference of 0.69 in favor of private institutions, ranking the quality of teaching higher in private institutions with a lower standard deviation, indicating more stable assessments. The average FAP for private is x=3.78 and SD=0.74 and public x=3.24 and SD=0.77, with an average difference of 0.54 in favor of private institutions, which showed greater adaptability by adapting to market demands, factors that reflect the functional orientation of the curriculum. The average HEIR for private is x=4.07 and SD=0.54 and public x=3.07 and SD=0.82, with an average difference of 1.00, representing the highest difference between institutions from all variables treated. According to this result, public institutions do not have an orientation to invest sustainably in modern infrastructure, reporting a lack of laboratories, functional spaces for learning, digitalization, etc., a situation that reduces the capacity for providing quality. The average EY for private is x=3.58 and SD=0.58 and public x=3.03 and SD=0.61, with an average difference of 0.55 in favor of private institutions. Such perception regarding employability is as an accumulated result of all the factors discussed above, where in each of them private institutions had the highest score. The average LMD for private is x=3.75 and SD=0.61 and public x=3.26 and SD=0.79, with an average difference of 0.49 in favor of private institutions which are perceived to have a higher match to the demands of the labor market.

**Table 9.** Descriptive results for private and public institutions.

Private				Public			
	N	Mean	Std. Deviation		N	Mean	Std. Deviation
SPH	130	3.6150	0.55325	SPH	170	3.3975	0.69147
CUB	130	3.4325	0.59761	CUB	170	2.8613	0.84833
TQ	130	3.7350	0.47250	TQ	170	3.0513	0.70946
FAP	130	3.7800	0.73879	FAP	170	3.2400	0.77038
HEIR	130	4.0700	0.54712	HEIR	170	3.0688	0.82438
EY	130	3.5780	0.58296	EY	170	3.0277	0.60573
LMD	130	3.7550	0.61132	LMD	170	3.2675	0.79434
RTI	130	2.1950	0.49439	RTI	170	2.2500	0.77896

**Note:** SPH = Study programs in higher education, CUB = Collaboration between the university and businesses, TQ = Teaching quality, FAP = Flexibility of Academic Programs, HEIR = Higher Education Infrastructure and Resources, LMD = Labor market demands, RTI = Role of technology and innovation, EY = Employability of youth.

*Source: Authors’ calculations based on survey data*. ^46^

The evident differences between private and public institutions present structural, strategic and managerial problems of public higher education in Kosovo. One factor that private institutions stand better in each component is because of competition and performance that has made their orientation towards matching the demands of the market and more functional preparation of students.

To determine whether the differences identified between private and public institutions are significant, a t-test was used for all variables included in the model. The results of the t-test (t (298) = –2.737, p = 0.007) for SPH show that the difference in the mean is significant, in which case it is confirmed that private institutions are marginally (0.23) better than public ones in terms of study programs. For the CBU variable, the results of the t-test (t (298) = –6.025, p < 0.05) prove that the difference between private and public institutions is significant, with private institutions being more structured and effective in the labor market. Similarly, the results of the t-test for TQ (t (298) = –8.716, p < 0.05) confirm that the difference in the mean of 0.68 in favor of private institutions is significant, which reinforces the presence of teaching standards. The results of the t-test for FAP (t (298) = –5.801, p < 0.05) also confirm the fact that private institutions offer more opportunities for curriculum adaptation as the results were significant. The variable with the largest difference turned out to be HEIR, where the results of the t-test (t (298) = –10.991, p < 0.05) confirm that the difference in the mean of 1.00 is significant, presenting a deep structural inequality between private and public institutions. The dependent variable EY also referred to the t-test (t (298) = –7.511, p < 0.001), identifies a statistically significant difference of 0.55 in favor of the private sector, a result which summarizes the cumulative impact of other factors addressed in the research, making private institutions have a higher affinity to prepare students for the labor market.

**Table 10.** T-test for differences between private and public institutions in the dimensions of higher education and employability.

		Levene's Test for Equality of Variances		t-test for Equality of Means						
		F	Sig.	t	df	Sig. (2-tailed)	Mean Difference	Std. Error Difference	95% Confidence Interval of the Difference	
									Lower	Upper
SPH	Equal variances assumed	4.966	0.027	-2.737	298	0.007	-0.21750	0.07946	-0.37388	-0.06112
	Equal variances not assumed			-2.946	240.924	0.004	-0.21750	0.07383	-0.36294	-0.07206
CBU	Equal variances assumed	13.411	0.000	-6.025	298	0.000	-0.57125	0.09481	-0.75783	-0.38467
	Equal variances not assumed			-6.746	265.107	0.000	-0.57125	0.08467	-0.73797	-0.40453
TQ	Equal variances assumed	14.882	0.000	-8.716	298	0.000	-0.68375	0.07845	-0.83813	-0.52937
	Equal variances not assumed			-9.922	274.479	0.000	-0.68375	0.06891	-0.81942	-0.54808
FAP	Equal variances assumed	0.369	0.544	-5.801	298	0.000	-0.54000	0.09308	-0.72319	-0.35681
	Equal variances not assumed			-5.883	205.668	0.000	-0.54000	0.09179	-0.72097	-0.35903
HEIR	Equal variances assumed	21.221	0.000	-10.991	298	0.000	-1.00125	0.09110	-1.18053	-0.82197
	Equal variances not assumed			-12.524	275.026	0.000	-1.00125	0.07995	-1.15864	-0.84386
EY	Equal variances assumed	0.127	0.422	-7.511	298	0.000	-0.55033	0.07327	-0.69453	-0.40614
	Equal variances not assumed			-7.608	205.013	0.000	-0.55033	0.07234	-0.69296	-0.40771

**Note:** SPH = Study programs in higher education, CUB = Collaboration between the university and businesses, TQ = Teaching quality, FAP = Flexibility of Academic Programs, HEIR = Higher Education Infrastructure and Resources, LMD = Labor market demands, RTI = Role of technology and innovation, EY = Employability of youth.

*Source: Authors’ calculations based on survey data*. ^46^

To measure the difference in the employability of young people (EY) depending on the three variables Level of education (LE), Grade point average (GPA), Field of study (FS), Working experience (WE). One Way ANOVA was performed. Given that the results for LE (F(2,297)=1.850; sig=0.159>0.05) and the results for GPA (F(3,296)=1.811; sig=0.145>0.05) are not statistically significant, we conclude that there is insufficient evidence that the employability of young people differs depending on the level of education and that the level of the average grade is not a significant indicator of employability. Unlike the two variables above, field of study (FS) (F (8,291) =10.043; sig=0.000) presents a statistically significant result, which means that the perception of employability varies depending on the field of study. Post Hoc tests were used to identify fields of study that have differences in employability

**Table 11.** Results of One-way ANOVA.

Employability of youth		Sum of Squares	Df	Mean Square	F	Sig.
LE	Between Groups	1.561	2	0.780	1.850	0.159
	Within Groups	125.291	297	0.422		
GPA	Between Groups	2.286	3	0.762	1.811	0.145
	Within Groups	124.566	296	0.421		
FS	Between Groups	27.445	8	3.431	10.043	0.000
	Within Groups	99.407	291	0.342		
Total		126.852	299			

**Note:** LE = Level of education, GPA = Grade point average, FS = Field of study, WE = Working experience.

*Source: Authors’ calculations based on survey data*. ^46^

.

Since the ANOVA results highlighted statistically significant differences regarding the field of study, through Post Hoc Tukey HSD we identify in detail the fields of study between which there are significant differences. The results of the Tukey HSD test present three homogeneous Subsets, classifying them according to the average of employability. In the first subset, the fields of study with the lowest average are emergency management). These fields show lower average scores in perceived employability and self-reported labor market alignment. In the second subset, fields such as legal science, economics and business, education and teaching, and social sciences and humanities are grouped, which although they do not have a negative perception do not offer the same level of security in relation to employability compared to technical fields. In subset three, engineering and architecture, and especially computer science and information technology, are grouped in fields that present the highest average and statistically significant difference. These findings represent a higher compatibility through the competencies acquired during the study and the demands of the labor market, mainly the technology and engineering sector.

The findings of this test report that the field of study is a critical factor in terms of employability, where graduates from technology and engineering-related fields report higher levels of perceived employability and more favorable self-reported labor market outcomes after graduation. This fact shows the need for strategic reform of the academic offer to achieve a match between the offer of higher education and the demands of the labor market.

**Table 12.** Tukey HSD test.

	Field of study	N	Subset for alpha = 0.05		
			1	2	3
Tukey HSD ^a^ ^,^ ^b^	Agriculture and Environmental Sciences	27	2.6833		
	Arts and design	23	2.8667	2.8667	
	Other	12	2.9333	2.9333	
	Legal science	39	3.0327	3.0327	
	Economics and business	50	3.2152	3.2152	
	Education and teaching	29	3.2762	3.2762	
	Social sciences and humanities	45	3.3238	3.3238	
	Engineering and architecture	31		3.7704	3.7704
	Computer science and information technology	44			4.3944
	Sig.		0.004	0.001	0.0490

*Source: Authors’ calculations based on survey data*. ^46^

Means for groups in homogeneous subsets are displayed.

^a^ Uses Harmonic Mean Sample Size = 6.482.

^b^ The group sizes are unequal. The harmonic mean of the group sizes is used. Type I error levels are not guaranteed.

Table 13 presents the hypotheses and sub-hypotheses raised in the research, along with the tests used to validate the hypotheses.

**Table 13.** Verification of hypothesis.

Alternative Hypothesis		Statistical test used	Result
**H**_**1**_ **: Higher education has a statistically significant impact on graduates’ perceived employability in Kosovo**		Simple linear regression	Accepted
**H**_**1a**_	Study programs in higher education have a statistically significant impact on graduates’ perceived employability.	Pearson’s correlation & Simple linear regression	Accepted
**H**_**1b**_	Collaboration between the university and businesses has a statistically significant impact on graduates’ perceived employability.	Pearson’s correlation & Simple linear regression	Accepted
**H**_**1c**_	Teaching quality has a statistically significant impact on graduates’ perceived employability.	Pearson’s correlation & Simple linear regression	Accepted
**H**_**1d**_	Flexibility of Academic Programs has a statistically significant impact on graduates’ perceived employability.	Pearson’s correlation & Simple linear regression	Accepted
**H**_**1e**_	Higher Education Infrastructure and Resources have a statistically significant impact on graduates’ perceived employability.	Pearson’s correlation & Simple linear regression	Accepted
**H**_**2**_ **: There is a statistically significant difference in the level of graduates’ perceived employability depending on the type of higher education institution (public or private)**		T-test	Accepted
**H**_**3**_ **: There is a statistically significant difference in the level of graduates’ perceived employability depending on the graduate profile characteristics**		ANOVA & Post Hoc test	Accepted
**H**_**3a**_	There is a statistically significant difference in the graduates’ perceived employability based on their level of education.	ANOVA	Rejected
**H**_**3b**_	There is a statistically significant difference in the graduates’ perceived employability based on their grade point average.	ANOVA	Rejected
**H**_**3c**_	There is a statistically significant difference in the graduates’ perceived employability based on their field of study.	ANOVA & Post Hoc test	Accepted
**H**_**4**_ **: External drivers of employability have a statistically significant impact on the graduates’ perceived employability**		Simple linear regression	
**H**_**4a**_	Labor market demand has a statistically significant impact on the graduates’ perceived employability.	Simple linear regression	Accepted
**H**_**4b**_	Role of technology and innovation has a statistically significant impact on the graduates’ perceived employability.	Simple linear regression	Rejected

## Limitations of the Study

This study should be interpreted considering several limitations related to research design and the data used. The analysis is based on self-reported information collected from graduates through a structured questionnaire. As a result, the findings mainly reflect respondents’ perceptions of the match between higher education labor market requirements, which may be influenced by individual experiences and subjective assessments. In addition, the concept of employability in this study is operationalized as perceived employability. The measurement relies on graduates’ own assessments of their readiness for labor market integration and the extent to which their skills match employers’ expectations. Consequently, the analysis does not include objective indicators of employment outcomes, such as current employment status, income levels, or long-term career progression. Another limitation is related to the cross-sectional nature of research design. Since the data were collected at a single point in time, statistical analyses identify relationships and associations between variables rather than causal effects. For this reason, the findings should be interpreted as indicative of patterns in graduates’ perceptions rather than as evidence of direct causal relationships between dimensions of higher education and employability outcomes. Finally, although the sample was proportionally constructed to reflect the distribution of graduates across public and private higher education institutions in Kosovo, the results should be interpreted primarily within the specific institutional and labor market context of the Kosovo higher education system.

## Conclusions

The results indicate a fragmentation between higher education provision and labor market demands, where even through some dimensions present positive responses, gaps are evident in the integration of practical skills, university-industry collaboration, and the inclusion of technology and innovation in study programs, suggest an uneven match between higher education provision and labor market demands. This leads to graduates who are not ready to face the challenges posed by the workplace. This paper also presents a significant difference between public and private HE institutions in Kosovo in the context of their preparation for the labor market. Private institutions were found to perform better compared to public institutions in all descriptive dimensions of HE such as study programs (SPH), teaching quality (TQ), university-industry collaboration (CUB), flexibility of academic programs (FAP), infrastructure and resources (HEIR) and labor market demands. This conclusion is supported by statistical inferential tests, demonstrating statistically significant differences.

The latent structures presented by the variables (SPH, CUB, TQ, FAP, HEIR, LMD) were also confirmed by factor analysis, where according to consistent results these variables represent important factors in the model of labor market preparation. The values of KMO (.889) and Bartlett’s Test, where p<0.05 are indicators of high suitability of the data for factor analysis. The association between these factors and perceived employability is also demonstrated through Pearson correlation and simple linear regression where LMD (R ^2^=.390) and FAP (R ^2^=.367) showed the strongest statistical associations with youth employability (EY) followed by TQ and HEIT. The RTI dimension did not show a statistically significant association with EY in the regression model, despite its theoretical importance in the literature. This finding suggests that, although technology and innovation are widely recognized as important in higher education, their empirical relationship with perceived employability was not confirmed in the present model. This result was the same for private and public institutions, showing the need for intervention to increase compatibility with technological developments in the market.

According to the results of the t-tests, a statistically significant difference was identified between private and public institutions regarding all variables of the model, where the most pronounced difference resulted in infrastructure and resources, where public institutions do not provide technical support and modern teaching environments. This situation will not only reflect on the quality of learning but will also be an indicator of a decrease in the potential for the development of practical skills, further increasing the gap between the labor market and higher education. Also, the results of One-way ANOVA showed that the field of study (FS) is an important factor in employability, where graduates of engineering, architecture, computer science and information technology have an advantage in employability in relation to fields such as agriculture, arts, legal sciences and social fields. This fact represents the compliance of these fields with the demands of the labor market, as a result requiring a redesign of the academic offer in accordance with current technological and economic trends. This research is an indicator that higher education in Kosovo is characterized by two relatively opposed realities, where the orientation of private institutions is towards competition and the market, while graduates from public institutions reported less favorable evaluations across several dimensions, suggesting institutional challenges related to flexibility, responsiveness and resource provision. These findings suggest the need for targeted improvements in public institutions, particularly in curriculum flexibility, infrastructure, and cooperation with labor market actors.

## Recommendations

Recommendations are grouped by stakeholders and their role in the provision of services, skills and competences to students in Kosovo. For more clarity the main groups of stakeholders were grouped as government authorities, higher education institutions, and industry. Dynamic developments that characterize the labor market and the slow reaction by the HEIs could result in furthering the mismatch between higher education provision and labor market demand. Based on the empirical results, the following recommendations are suggested, with the goal to contribute to improving the grave situation regarding higher education provision with labor market demands:

**Government authorities:**

• Engage in designing strategic investments through a medium-term investment plan in higher education for provision of specialized laboratories for applied research and practical learning, creation of electronic libraries, professional software and the creation of spaces for project-based learning to address the structural difference between private and public higher education institutions;
• Create a permanent observatory that aims to (i) measure the impact of HE on labor market demands and (ii) serve as a national monitoring mechanism focusing on the performance of HE institutions in relation to the employability of graduates;
• Implement equal accreditation and monitoring processes for both private and public institutions to ensure the same standards of academic performance and provide for an environment that mitigates monopolistic position and mobilizes public HE institutions towards meeting the student and labor market needs;

### Higher education institutions

• Reconceptualize study programs to develop practical skills oriented towards a dynamic labor market. Accordingly, it is recommended to take measures towards a systematic process that enables the revision of curricula by supporting the competency-based approach, through the involvement of industry in the design of curricula;
• Develop sustainable mechanisms that ensure cooperation between higher education institutions and industry, including creation of a permanent functional structure of stakeholders in lieu of the current industrial boards to include also program management, students, alumni, teachers, and community representatives in the curriculum development process;
• Institutionalize agreements for professional and work-based learning practices and the creation of partnerships aimed at developing applied projects that facilitate the transfer of theoretical knowledge into practice;
• Reorient the teaching methodology for the development of competencies, offering methods based on real-world problem solving, simulation or integrated projects and the inclusion of technology in the learning process;
• Provide consistent training for academic staff with the aim of developing innovative pedagogic practices;
• Strengthen the flexibility component of study programs, as it is one of the most important determinants of employability. This can be achieved through the promotion and design of flexible modules that enable students to have more personalized curricula and the inclusion of practical work in the assessment;
• Create internal mechanisms aimed at observing market trends and making regular reassessments regarding their academic offer;
• Orient students towards fields that enable easier and more sustainable employability, by intensifying information campaigns on market demand through a market research mechanism. This labor market research should be oriented towards the demand of employers for fields of interest by reinforcing current findings;
• Engage in building organic partnerships with industries engaged in similar fields as academic disciplines to inform and facilitate curriculum design, planning and implementation of practical learning experiences, and for designing and implementation of joint development and applied research projects;

**Industry:**

• Use professional organizations and chambers of commerce as channels for facilitating communication and cooperation with higher education institutions;
• Apply a more proactive approach in promoting their interests and needs for expertise and transfer of know-how with and from higher education institutions;
• Engage in partnerships with relevant universities as a best means for ensuring their competitiveness through innovative processes in production and provision of services;

## Ethics and consent statement

Ethical approval for this research was granted by the Ethics Committee of AAB College, Prishtina, Kosovo (Ref. No. 262/2025, 22 May 2025). All participants provided electronic written informed consent prior to participation via Microsoft Forms. The survey was anonymous, and no identifying data was collected. No minors participated. The study fully complied with the General Data Protection Regulation (GDPR, Regulation (EU) 2016/679) and institutional data-protection standards.

## Data Availability

The dataset supporting the results of this article is openly available in Figshare at https://doi.org/10.6084/m9.figshare.30334438. ^46^ Data are available under the terms of the Creative Commons Zero “No rights reserved” ( CC0 1.0 Public Domain Dedication). It includes the full questionnaire, raw data, and SPSS analysis files used in this research. Supplementary materials, including the questionnaire used for the employability assessment and coding manual, are openly available in the same Figshare repository at: https://doi.org/10.6084/m9.figshare.30334438. ^46^ Data are available under the terms of the Creative Commons Zero ( CC0 1.0 Public Domain Dedication).
