# Supplementary material for: The impact of higher education on youth employability in Kosovo: Key determinants affecting the interface between higher education provision and labor market needs
Source: Open Res Eur. 2026 May 11;6:15. Originally published 2026 Jan 6. [Version 2] doi: 10.12688/openreseurope.21616.2 (PMC13221626; doi:10.12688/openreseurope.21616.2)
Supplement: Peer review history

# Reviewer report: Reviewer response for version 2

**DOI:** 10.21956/openreseurope.25323.r74014

**Published:** 2026-05-29

**Authors:** Adilah Hisa

**Affiliations:** 1 University of Brunei Darussalam (Ringgold ID: 37597), Bandar Seri Begawan, Brunei-Muara District, Brunei

No further comments.

If applicable, is the statistical analysis and its interpretation appropriate?

Partly

Is the study design appropriate and is the work technically sound?

Partly

Is the work clearly and accurately presented and does it engage with the current literature?

Partly

Are the conclusions drawn adequately supported by the results?

Partly

Are sufficient details of methods and analysis provided to allow replication by others?

Partly

Are all the source data and materials underlying the results available?

Yes

Reviewer Expertise:

Innovation management, leadership, employability, youth development, human capital development, knowledge management, organizational behavior, and supply chain management,

I confirm that I have read this submission and believe that I have an appropriate level of expertise to confirm that it is of an acceptable scientific standard.

# Reviewer report: Reviewer response for version 2

**DOI:** 10.21956/openreseurope.25323.r74015

**Published:** 2026-05-29

**Authors:** Balwant Singh Mehta

**Affiliations:** 1 Institute for Human Development, New Delhi, New Delhi, India

I have reviewed the authors’ responses and the summary of revisions. The authors have satisfactorily addressed my earlier concerns, particularly regarding the definition of employability, clarification of the methodology, alignment of conclusions with the statistical findings, treatment of causal language, and acknowledgement of study limitations.

 I am satisfied with the revisions made and believe that the manuscript has improved sufficiently. I am therefore happy to amend my previous status and recommend that the article be updated to Approved.

If applicable, is the statistical analysis and its interpretation appropriate?

Partly

Is the study design appropriate and is the work technically sound?

Partly

Is the work clearly and accurately presented and does it engage with the current literature?

Partly

Are the conclusions drawn adequately supported by the results?

Partly

Are sufficient details of methods and analysis provided to allow replication by others?

Partly

Are all the source data and materials underlying the results available?

Yes

Reviewer Expertise:

Employment, Unemployment, Inequality, Gender, and Education

I confirm that I have read this submission and believe that I have an appropriate level of expertise to confirm that it is of an acceptable scientific standard.

# Reviewer report: Reviewer response for version 2

**DOI:** 10.21956/openreseurope.25323.r74013

**Published:** 2026-05-20

**Authors:** Theodor Leiber

**Affiliations:** 1 Evaluationsagentur Baden-Württemberg, Heidelberg, Germany

No further comments

If applicable, is the statistical analysis and its interpretation appropriate?

Yes

Is the study design appropriate and is the work technically sound?

Yes

Is the work clearly and accurately presented and does it engage with the current literature?

Yes

Are the conclusions drawn adequately supported by the results?

Yes

Are sufficient details of methods and analysis provided to allow replication by others?

Yes

Are all the source data and materials underlying the results available?

Yes

Reviewer Expertise:

evaluation and accreditation; impact analysis; performance indicators; digital competencies; learning and teaching spaces; student pathways through education and training and to the labour market; social science methodologies; ethics

I confirm that I have read this submission and believe that I have an appropriate level of expertise to confirm that it is of an acceptable scientific standard.

# Reviewer report: Reviewer response for version 1

**DOI:** 10.21956/openreseurope.23385.r69200

**Published:** 2026-03-06

**Authors:** Balwant Singh Mehta

**Affiliations:** 1 Institute for Human Development, New Delhi, New Delhi, India

- Key concepts, including employability, employment, and labor market demand, are used interchangeably throughout the manuscript. The study assesses perceived employability using Likert-scale responses rather than actual employment status. For example, the Methods section indicates that employability is measured via a structured questionnaire with Likert scales (p. 6–7). It is recommended that the authors clearly define “employability” (distinguishing perceived readiness from actual employment outcomes) and differentiate it from the employment rate.

 

- Although the literature review is comprehensive, it is repetitive and relies predominantly on studies published before 2021. For a 2026 publication, it is necessary to integrate more recent empirical research (2021–2025) on graduate employability and skills mismatch.

 

- On page 9, the manuscript asserts that “there is still no coherence between the objectives of higher education and the labor market.” This statement appears interpretive rather than directly supported by the data. The authors should explicitly connect this conclusion to specific statistical findings.

 

- The quantitative design is appropriate, and statistical tools are correctly applied (KMO = 0.889; Bartlett p < .001; Durbin–Watson = 2.073). However, while the study claims to use “simple random selection” (p. 6), it does not specify how graduates were contacted, the response rate, or whether non-response bias was assessed. Although the five higher education dimensions are described, the manuscript does not clarify whether the items were adapted from validated scales, whether a pilot study was conducted, or whether the Likert items were pretested. Additionally, presenting hypotheses within the Methods section (p. 7) disrupts the logical flow; these should be introduced after the literature review.

 

- The statistical formulas and regression outputs are detailed (Tables 3–8), and the dataset is publicly available via Figshare. However, replication requires a full questionnaire appendix, exact Likert wording, coding scheme, and handling of missing data. These details should be summarized within the manuscript, not only referenced externally.

 

- The dataset and SPSS files are publicly available via Figshare under a CC0 license (Data availability section, p. 20), which represents a significant strength of the manuscript.

 

- The statistical techniques employed are appropriate and correctly executed. However, some interpretations overstate causality, as regression analysis demonstrates association rather than causal impact. Technology and innovation (RTI) was excluded due to p = 0.18 (Table 8), yet its importance is discussed in the conclusions. This inconsistency should be addressed. The authors are advised to avoid causal language, replacing terms such as “impact” with “association” unless causality is empirically justified. The theoretical versus empirical role of RTI should also be clarified.

 

- The conclusions generally align with the regression and ANOVA findings (for example, the strong roles of LMD and FAP, and field-of-study differences as shown in Table 12). However, the conclusion section functions more as an extended discussion, and there is no explicit limitations section. Structural criticisms of public universities (p. 19) extend beyond the empirical evidence, which is based on perceptions. The manuscript would benefit from clearly separated results, discussion, conclusion, and limitations sections. A dedicated limitations section should address issues such as self-reported data, cross-sectional design, and perception bias. Normative claims not directly supported by data should be avoided.

**Recommendation: Major Revision Required**

If applicable, is the statistical analysis and its interpretation appropriate?

Partly

Is the study design appropriate and is the work technically sound?

Partly

Is the work clearly and accurately presented and does it engage with the current literature?

Partly

Are the conclusions drawn adequately supported by the results?

Partly

Are sufficient details of methods and analysis provided to allow replication by others?

Partly

Are all the source data and materials underlying the results available?

Yes

Reviewer Expertise:

Employment, Unemployment, Inequality, Gender, and Education

I confirm that I have read this submission and believe that I have an appropriate level of expertise to confirm that it is of an acceptable scientific standard, however I have significant reservations, as outlined above.

# Response

**Published:** 2026-03-08

**Authors:** Arbëresha Qerimi

**Affiliations:** Economics, AAB College, Prishtina, Prishtina, Kosovo (Serbia and Montenegro)

Dear Dr. Balwant Singh Mehta, We sincerely thank you for the time and attention you have devoted to reviewing our manuscript. Your comments and suggestions have been very valuable and have helped us reflect more deeply on several important aspects of the study. Your observations have contributed to improving the clarity of the argumentation, the methodological transparency, and the presentation of the results. Taking your comments into account, we have carefully revised the manuscript and have integrated improvements in several important parts of it. The literature review has been updated by incorporating more recent empirical studies on graduate employability and the mismatch between skills and labor market demands, so that the theoretical discussion better reflects contemporary developments in this field. In addition, the methodology section has been expanded to provide greater clarity regarding the study design, the data collection process, and the structure of the measurement instrument. In this section, it has been clarified that the dimensions and items of the questionnaire were developed by the authors based on the existing literature and were preliminarily tested through a pilot phase before the start of data collection. Clarifications have also been added regarding the use of the Likert scale, the coding of variables, and the handling of missing data. In order to increase transparency and the possibility of replicating the study, the full questionnaire has been included in the appendix. In the revised version, we have also reconsidered the interpretation of the results in order to avoid formulations that might suggest causal relationships. Instead, the findings have been interpreted more carefully as statistical relationships or associations between variables. In this context, the role of the technology and innovation variable (RTI) has also been clarified, emphasizing that although it is treated as an important factor in the literature, in the empirical analysis of this study it did not appear as statistically significant in the regression model. The structure of the analytical sections has also been revised in order to strengthen the link between the empirical results and the conclusions of the study. At the same time, formulations that could be interpreted as normative assessments have been moderated, so that the interpretation remains consistent with the perceptual nature of the data used in the analysis. The manuscript now also includes a dedicated section on the limitations of the study, where the methodological limitations related to the use of self-reported data, the measurement of employability as perceived employability, the cross-sectional design of the study, as well as the specific context of the higher education system in Kosovo are discussed.

 We hope that these revisions have adequately addressed your comments and have contributed to improving the quality and clarity of the manuscript. We thank you once again for the valuable comments and suggestions that have helped improve this study.

# Reviewer report: Reviewer response for version 1

**DOI:** 10.21956/openreseurope.23385.r69197

**Published:** 2026-02-26

**Authors:** Theodor Leiber

**Affiliations:** 1 Evaluationsagentur Baden-Württemberg, Heidelberg, Germany

The article is very well written and clearly structured. The objectives the article mentions are very relevant and timely and achieved by the findings. The methods used are clearly described and fully appropriate to research interest, questions and hypotheses. In particular, the statistical analyses are quite elaborate. 

 Against this background, I only have a few points and suggestions that might be considered by the authors: 

 The concepts "employment" and "employability" (and also "employment rate") are notoriously confounded in debates and literature, while "employment" and "employability" denote quite different issues. In particular, “employability” is hard to measure, to say the least, while this is far less the case for “employment” (simply: “having a job”). Hence, it might be checked where “employability” used in the paper (including its title) should be replaced by “employment” or “employment rate”.

 It is true that the quantitative data used are all based on the respondents’ qualitative perceptions quantified through Likert scales. This might be made a bit more explicit for the reader by mentioning it more explicitly, e.g., on the first page under "Methods".

 On page 4, left column towards the end of the 4 ^th^ paragraph: “theory based teaching” should be corrected to “theory-based teaching”.

 Figure 1 and Figure 2 (not their figure captions) must be exchanged, see p. 5, right column, last paragraph as well as pp. 6 and 7.

 Page 5, right column, last paragraph: the text reads as if Figure 1 would also show the number of students (and not only graduates and unemployed graduates) over the respective years. However, the number of students is not shown in the graph, i.e., please amend the graph or change the text.

 The caption of the figure now on page 7 should read “Number of graduated students and of unemployed graduates from higher education”. Same change should be made in the legend of this figure. When the text refers to this figure (e.g., p. 5, right column, last paragraph), using “employability” is not appropriate because this figure shows the number of unemployed graduates from higher education (and not their employability, whatever it may be).

 The caption of Table 1 talks about “the last five years” which is no longer correct as we have 2026 already. Better: replace by “Number of graduate students from 2019/20 to 2023/24”.

 Objective 1 (and further on) talks about “the employability LEVEL of graduates”. To me, it would sound more appropriate to talk about the “employment rate” instead of “employment level”.

 Page 9, right column, 2 ^nd^ paragraph: In “over the past FIVE years” “five” is probably not (no longer) correct. 

 Page 9, right column, 3 ^rd^ paragraph, 4 ^th^ line: “with of the” should be corrected.

 Page 9, right column, 3 ^rd^ paragraph, 4 ^th^ line: How do you know that “there is still no coherence between the objectives of higher education and the labor market. As a result, higher education graduates appear … the challenges of the labor market.” (I am not opposing the hypothesis but ask for its empirical justification.)

 Figs. 4 and 5: The figure captions are not immediately clear. Suggestion: replace by “Respondents' agreement with the variables …”. This would also be in better agreement with the respective figure legends.

 Page 11, last ten lines: I assume that the hypotheses formulated their originate from the survey you carried out …

 Page 19, 2 ^nd^ paragraph, line 11: „HEIT” should be replaced by “HEIR”.

 Page 19, 3 ^rd^ paragraph, line 11: „FS” should be replaced by “(FS)”.

 Page 19, 3 ^rd^ paragraph, lines 20 ff.: Please check whether the mentioned negative characteristics can be attributed to public universities based on the available research results (data). How do the different funding and personnel strategies of private and public universities compare?

If applicable, is the statistical analysis and its interpretation appropriate?

Yes

Is the study design appropriate and is the work technically sound?

Yes

Is the work clearly and accurately presented and does it engage with the current literature?

Yes

Are the conclusions drawn adequately supported by the results?

Yes

Are sufficient details of methods and analysis provided to allow replication by others?

Yes

Are all the source data and materials underlying the results available?

Yes

Reviewer Expertise:

evaluation and accreditation; impact analysis; performance indicators; digital competencies; learning and teaching spaces; student pathways through education and training and to the labour market; social science methodologies; ethics

I confirm that I have read this submission and believe that I have an appropriate level of expertise to confirm that it is of an acceptable scientific standard.

# Response

**Published:** 2026-03-06

**Authors:** Fidan Qerimi

**Affiliations:** AAB College, Prishtina, Prishtina, Kosovo (Serbia and Montenegro)

Dear Dr. Theodor Leiber, Thank you very much for your careful reading of our manuscript and for your constructive and insightful comments. We sincerely appreciate the time and expertise you have invested in reviewing the paper. Your observations have been very valuable in helping us further improve the clarity, precision and overall quality of the manuscript. We are pleased to inform you that all the comments and suggestions you provided have been carefully considered and addressed in the revised version of the manuscript. In particular, we have reviewed the use of the concepts related to employability, employment and employment rate throughout the manuscript and made the necessary adjustments to ensure conceptual clarity and consistency. Where appropriate, the terminology has been corrected in the title, objectives and discussion in order to better reflect the nature of the variables analysed. Following your suggestion, we have also clarified in the Methods section that the empirical data are based on respondents’ perceptions measured through Likert-scale responses, making this aspect more explicit for the reader. In addition, several technical and textual corrections have been made throughout the manuscript. These include the correction of typographical issues (such as “theory-based teaching” and other minor language corrections), the exchange of Figures 1 and 2, revisions to figure captions and legends, clarification of the description of the figures in the text, and corrections in tables and references to years where necessary. Furthermore, the captions of Figures 4 and 5 have been reformulated to more clearly indicate that they present respondents’ agreement with the analysed variables. We have also corrected the abbreviations and formatting issues you indicated (e.g., HEIR, FS) and revised the relevant sections of the discussion to better justify statements related to the relationship between higher education outcomes and labour market demands. Finally, the section discussing differences between public and private higher education institutions has been reviewed to ensure that all statements are consistent with the available research evidence and data presented in the study. Once again, we would like to express our sincere gratitude for your constructive feedback. Your comments have significantly contributed to improving the clarity and academic quality of the manuscript.

 Kind regards,

 Arberesha and co-authors

# Reviewer report: Reviewer response for version 1

**DOI:** 10.21956/openreseurope.23385.r69196

**Published:** 2026-02-26

**Authors:** Adilah Hisa

**Affiliations:** 1 University of Brunei Darussalam (Ringgold ID: 37597), Bandar Seri Begawan, Brunei-Muara District, Brunei

Summary: The manuscript addresses a relevant topic but requires major revision. Engagement with recent literature is limited, methodological details are insufficient for replication, and sections (results, discussion, conclusion) lack clear separation. Statistical analyses are appropriate but overly descriptive. Conclusions are partially supported but require stronger theoretical integration.

 Introduction

 The introduction identifies a clear and relevant problem: the mismatch between higher education outcomes and labor market demands. The aims of the study are stated clearly, including measurement of the impact of higher education on employability and exploration of HEI ownership as a factor.

-
  - The introduction is very long and repetitive. Many sentences reiterate similar points about employability, skills gaps, and labor market mismatch.
  - For a study claiming contemporary relevance, recent literature (2021–2025) should be more extensively integrated.
  - Terms like *employability*, *skills gap*, and *practical competencies* are used frequently but are not conceptually defined or operationalized.
  - While the introduction describes the mismatch problem in Kosovo, the specific gap in the literature is not clearly articulated. For instance:
    - What existing studies exist on Kosovo graduates’ employability?
    - What has not been addressed (e.g., role of private vs. public HEIs, impact of program design, or regional comparisons)?
  - The introduction hints at societal and economic importance but does not explicitly connect the study’s findings to broader implications for policy, curriculum design, or regional development.

Literature Review:

- The review identifies key themes, including employability, skills mismatch, and the role of higher education in preparing graduates for the labor market.
- It is very lengthy and repetitive, with multiple sentences reiterating similar points about competencies, labor market dynamics, and practical vs. theoretical skills.
- Key concepts (e.g., *employability*, *transferable skills*, *competency mismatch*) are mentioned but not consistently defined or critically discussed.
- The Kosovo context is described in detail, but linkage to prior literature and theoretical frameworks is weak, and the specific research gap remains unclear.
- Many citations are older or general; recent empirical studies (2021–2025) on graduate employability and skills mismatch are underrepresented.

Comment on Methods:

 The Methods section provides useful details on the sample, data collection, and measurement instruments. However, the section currently intermixes hypothesis statements with methodological descriptions, which disrupts the logical flow and makes it harder for the reader to distinguish between study design and empirical objectives. For clarity and readability, the manuscript should present the study methods—including sample, instruments, and analysis techniques—separately, and report hypotheses in a distinct section (perhaps a subsection under literature review, where empirical evidence are presented in relation to the hypothesis statements).

- The overall method is clearly quantitative with a defined population and sample, but the description lacks sufficient detail about sampling procedures beyond “simple random selection” (e.g., how participants were contacted, response rate, potential non-response bias).
- The objectives and hypotheses are numerous (four objectives, four hypotheses) and appear disconnected from the earlier Introduction/Literature Review, which focused mainly on employability and skills mismatch. It is unclear how some hypotheses (e.g., external drivers of employability) are operationalized.
- The structure of the instrument is described, but the source or validation of the scales is missing. Were these adapted from prior studies or newly developed? How were the Likert items constructed and pre-tested?
- The section on statistical tests (Kolmogorov-Smirnov, Shapiro-Wilk, Pearson, Cronbach’s alpha, EFA) is out of place in the Methods narrative—it reads more like Results/Analysis justification rather than procedures. These should be presented clearly as part of the data analysis plan.
- The manuscript also lacks descriptions of the measurement items and the sources from which they were obtained. These information should be mentioned in the Method section.

Conclusions

- The section titled *Conclusions* reads more like a Discussion section, as it continues interpreting statistical findings, comparing public and private institutions, and elaborating on factor analysis results.
- A true Conclusion should concisely summarize the main findings, restate the study’s contribution, and highlight overall implications — without re-reporting detailed statistical outputs.

- The manuscript would benefit from clearer structural distinction between:
  - Results (statistical findings),
  - Discussion (interpretation and theoretical/practical meaning),
  - Conclusion (brief synthesis and contribution).
- The section provides statistical outputs (R² values, t-tests, ANOVA), but the discussion does not sufficiently reconnect the findings to prior literature review/empirical evidence – this should be done in discussion section.

Separation of Sections – The manuscript merges results, interpretation, and recommendations; consider structuring Discussions, Conclusions, Limitations, and Recommendations as distinct sections for clarity.

1. Missing Limitations – No explicit discussion of study limitations is provided; adding this would contextualize findings and prevent overgeneralization.
2. Overlap with Results – Some recommendations repeat results or statistical findings; streamlining these would improve readability.
3. Evidence vs. Interpretation – Empirical evidence (factor analysis, t-tests, ANOVA) should be presented separately from interpretive discussion for better flow.
4. Stakeholder Recommendations – While detailed, recommendations could be prioritized or summarized to enhance clarity and impact.

If applicable, is the statistical analysis and its interpretation appropriate?

Partly

Is the study design appropriate and is the work technically sound?

Partly

Is the work clearly and accurately presented and does it engage with the current literature?

Partly

Are the conclusions drawn adequately supported by the results?

Partly

Are sufficient details of methods and analysis provided to allow replication by others?

Partly

Are all the source data and materials underlying the results available?

Yes

Reviewer Expertise:

Innovation management, leadership, employability, youth development, human capital development, knowledge management, organizational behavior, and supply chain management,

I confirm that I have read this submission and believe that I have an appropriate level of expertise to state that I do not consider it to be of an acceptable scientific standard, for reasons outlined above.

# Response

**Published:** 2026-03-08

**Authors:** Arbëresha Qerimi

**Affiliations:** Economics, AAB College, Prishtina, Prishtina, Kosovo (Serbia and Montenegro)

Dear Dr. Adilah Hisa, Thank you for your careful reading of the manuscript and for the detailed and thoughtful comments you provided. Your observations were very helpful in identifying several aspects of the paper that required further clarification, particularly regarding the conceptual framing, the structure of the manuscript, and the transparency of the methodological description. We greatly appreciate the time and attention you devoted to reviewing our work. Following your comments, the manuscript has been revised in several important respects. The introduction and literature review have been substantially revised in order to reduce repetition and improve conceptual clarity. Additional recent empirical studies (2021–2025) on graduate employability and skills mismatch have been incorporated, and key concepts such as employability, skills mismatch, and practical competencies are now more clearly defined and discussed within the theoretical framework. The revised introduction also clarifies the research gap addressed by the study, particularly the limited empirical evidence on the relationship between higher education dimensions and graduates’ perceived employability in Kosovo, as well as the comparative perspective between public and private higher education institutions. Several revisions were also made to the methodological section in response to your comments. The description of the sampling procedure has been expanded, including additional clarification regarding the way participants were contacted, the response process, and the overall data collection procedure. The structure and development of the measurement instrument are now explained more clearly. Specifically, the dimensions and questionnaire items were developed by the authors based on the existing literature on graduate employability and higher education–labour market alignment, and the instrument was pretested through a pilot phase prior to the main data collection in order to ensure clarity and relevance of the items. Additional information has also been included regarding the measurement items, the use of Likert-scale responses, the coding of variables, and the statistical analysis procedures. These clarifications were added to improve the transparency of the methodological approach and facilitate potential replication of the study. In response to your comments on the structure of the manuscript, the analytical sections have been reorganized in order to create a clearer distinction between the presentation of empirical findings and their interpretation. The results are now presented more distinctly from the discussion, while the conclusion section has been revised to provide a more concise synthesis of the main findings and the contribution of the study. Furthermore, the manuscript now includes a dedicated section addressing the limitations of the study. This section discusses the limitations related to the use of self-reported data, the cross-sectional design of the research, and the measurement of employability as a perception of graduates rather than an objective labour market outcome. We believe that these revisions have significantly improved the clarity, structure, and methodological transparency of the manuscript. We are grateful for your constructive feedback, which has helped us strengthen the overall quality of the study.

 Kind regards, Arberesha and co-authors
